# Effect of Pulmonary Rehabilitation for Patients With Post-COVID-19: A Systematic Review and Meta-Analysis

**DOI:** 10.3389/fmed.2022.837420

**Published:** 2022-02-21

**Authors:** Huan Chen, Hangyu Shi, Xitong Liu, Tianheng Sun, Jiani Wu, Zhishun Liu

**Affiliations:** ^1^Department of Acupuncture, Guang'anmen Hospital, China Academy of Chinese Medical Sciences, Beijing, China; ^2^Tianjin University of Traditional Chinese Medicine, Tianjin, China; ^3^Beijing University of Chinese Medicine, Beijing, China; ^4^Department of Respiratory Disease, Beijing Hospital of Traditional Chinese Medicine, Beijing, China

**Keywords:** pulmonary rehabilitation, respiratory impairment, post-COVID-19 patients, systematic review, exercise capacity

## Abstract

**Background:**

Evidence increasingly suggested that impaired respiratory function remained in about 40% of patients with coronavirus disease 2019 (COVID-19) after discharge, jeopardizing their activities of daily living and quality of life (QoL) in a long term. Pulmonary rehabilitation (PR) can improve exercise capacity and QoL in individuals with chronic lung disease; however, evidence on the effect of PR for patients with post-COIVD-19 was scarce. This study aimed to conduct a systematic review and meta-analysis to evaluate the effect of PR on lung impairment for patients with post-COVID-19.

**Methods:**

Five databases were searched for all the published trials of PR for patients with post-COVID-19 from 2019 to October 2021. Data were extracted using a standardized form. The risks of bias of included studies were assessed using the Cochrane risk of the bias assessment tool. Data were synthesized where possible; otherwise, qualitative analysis was done.

**Results:**

Among 6,000 retrieved studies, 3 studies with 233 patients after COVID-19 were included. The pooled estimate of PR effect on 6-min walk test (6-MWT) (50.41, 95% CI 34.34 to 66.48; *p* < 0.0001) was in favor of the experiment group with clinical importance. It is found that PR could improve the symptom of dyspnea and QoL; however, its effect on pulmonary function test was inconsistent across studies. The risk of bias of included studies varied, with major concerns on the risk of blinding of participants and interventions performers.

**Conclusion:**

The review showed that PR could improve exercise capacity measured by 6-MWT among patients with mild-to-moderate lung impairment after COVID-19. The interpretation of effects on lung function, dyspnea, and QoL should be cautious due to inadequate and conflicting data reported across studies.

**Systematic Review Registration:**

https://www.crd.york.ac.uk/prospero/display_record.php?ID=CRD42021289562, identifier: CRD42021289562.

## Background

According to the WHO coronavirus disease 2019 (COVID-19) Dashboard (https://covid19.who.int/), at the end of December 2021 the global cumulative cases and deaths of COVID-19 reached over 300 million and 5.4 million, respectively. Evidence suggests that the lung is the organ most affected by COVID-19 with different pathophysiological events, including diffuse alveolar epithelium destruction, hyaline membrane formation, capillary damage and bleeding, alveolar septal fibrous proliferation, and pulmonary consolidation (1, 2). Lung impairment, such as restrictive ventilation disorders, compromised diffusion capability, and residual impaired lung function, was mostly reported by studies and could decrease activities of daily living (ADL) and quality of life (QoL) among survivors of COVID-19 (3). Mechanical ventilation (MV), the commonest life-saving modality in the intensive care unit (ICU) for patients with severe COVID-19, can limit mobility of diaphragm muscle and lead to prompt onset of impaired functions of respiratory muscles (4, 5). A systematic review on respiratory function in 380 patients with post-COVID-19 demonstrated that patients had altered respiratory function, and impaired diffusion capacity was observed in about 40% of patients at 1 to 3 months after discharge (6). A cohort study of 1,733 discharged patients with COVID-19 reported that the more severe patients were ill during the hospital stay, the worse impaired pulmonary diffusion capacity they suffered with, as well as fatigue or muscle weakness, sleep difficulty, and anxiety or depression, at 6 months after acute infection (7). Similarly, previous literatures on severe acute respiratory syndrome (SARS) and Middle East respiratory syndrome (MERS) suggested that patients may experience persistent respiratory impairment lasting for months or even years after being discharged (8, 9).

The American Thoracic Society (ATS) and the European Respiratory Society (ERS) statement on pulmonary rehabilitation (PR) concluded that PR can reduce dyspnea, increase exercise capacity, and improve QoL in individuals with chronic obstructive pulmonary disease (COPD), and may also result in meaningful short-term benefits in patients with interstitial lung diseases (ILD) (10, 11). Meanwhile, the study showed that early PR treatments can enhance physical outcomes and QoL among ICU survivors (12). A review suggested that a comprehensive rehabilitative approach comprising a multidisciplinary team offering cardiorespiratory, neuromuscular, and psychological interventions should be offered for patients with post-COVID-19 (13). However, most published evidence on the effect of PR for patients with post-COVID-19 were preliminary and no randomized controlled trials (RCTs) were even included in the previous review on this topic.

As the number of confirm COVID-19 cases and relevant publications are accumulating rapidly, we decided to conduct this systematic review to detect and describe the types of PR applied for patients with post-COVID-19, and to evaluate the effect of PR on lung impairment in order to provide useful information for practice in clinical and community settings.

## Methods

The systematic review was performed according to the Preferred Reporting Items for Systematic Reviews and Meta-Analyses (PRISMA) guidelines and has been registered at the International Prospective Register of Systematic Reviews (PROSPERO) with ID No. CRD42021289562 (14).

### Search Strategy

All published studies on PR for patients with post-COVID-19 from 2019 to October 2021 were searched in five databases, including PubMed, EMBASE, the Chinese Science and Technology Journal Full-text Database (CNKI), Wan Fang Data, and the Chinese Biomedical Literature Database (VIP). The key search terms included “post-Covid-19,” “lung,” “respiratory impairment,” “sequela,” “rehabilitation”, “randomized controlled trial,” etc. The tailored search strategy was applied to each database. Certain terms in Chinese were also adapted and searched in Chinese databases. A manual search of the references of included studies was also conducted to identify additional studies.

### Study Selection Criteria and Process

The studies were included if they: (1) focused on patients with pulmonary and functional impairment after COVID-19; (2) were RCTs, quasi-RCTs, or controlled trials; (3) were using PR intervention in experiment or control group. The definition of PR in this review followed the definition agreed by ATS/ERS in 2013, and intervention adopted by the studies could contain but not limited to exercise training, acupuncture and electrical stimulation, education, behavior change, etc.; (4) were reported at least one of the following outcomes measures: functional exercise capacity, e.g., 6-min walk test (6-MWT), etc.; dyspnea severity, e.g., dyspnea severity index (DSI), modified British Medical Research Council dyspnea score (mMRC), etc.; pulmonary function test (PFT), e.g., forced expiratory volume in 1 s (FEV1), forced vital capacity (FVC), FEV1/FVC, total lung capacity (TLC), diffusion capacity of the lungs for carbon monoxide (DLCO), and/or percentage against their predicted values (% predicted), etc.; QoL, e.g., the Short Form Health Survey-12 or 36 (SF-12, SF-36), EuroQuality-5Dimensions-3Levels questionnaire (EQ-5D-3L), etc.; anxiety or depression status, and adverse events of treatment (10).

The studies were excluded if they: (1) did not focus on patients with sequelae of COVID-19 infection; (2) did not focus on lung disease; (3) were using surgical interventions in any of the groups; (4) were cohort studies, case–control studies, cross-sectional studies, literature, and systematic reviews, etc.; (5) did not provide detailed data in original paper for further analysis.

After removal of duplications, titles and abstracts of identified studies were independently reviewed by two investigators with experience in the systematic review. Studies that were not relevant to the review were excluded. Then, full texts of the remaining studies were retrieved and reviewed by the two investigators against the eligibility criteria of the review. Any disagreements were solved by a senior supervisor.

### Data Extraction

The following information was extracted from included studies, including author name, year of publication, country, study design, sample size, inclusion criteria, ICU/MV history, co-morbidity, interventions of experiment and control groups, treatment regimen, frequency and follow-up, outcome measurements. Two investigators extracted data independently using a standardized form, and disagreements if any were solved by a senior supervisor.

### Assessment of Risk of Bias

The risk of bias of included trials was assessed according to the Cochrane risk of bias assessment tool on the following seven aspects: random sequence generation, allocation concealment, blinding of participants and personnel, blinding of outcome assessment, incomplete outcome data, selective reporting, and other bias. The risk of bias in each category was divided into three levels: low, unclear, and high risk and the assessment was conducted using software Review Manager 5.4.1.

### Data Analysis

Studies were categorized based on types of interventions. For the continuous variable, the mean difference (MD) of change before and after the intervention was used to measure treatment effect with 95% CI between the comparing groups. For dichotomous variables, the treatment effect between groups was presented as a risk ratio (RR) with 95% CI. When outcome data after the intervention, rather than data of change after intervention from baseline, was compared directly to assess the effect of the intervention, between-group difference was calculated and compared based on data provided by included studies. Meta-analysis was applied to synthesize outcome data where study designs and outcome measures were comparable based on clinical criteria. Whether a fixed or a random effect model should be adopted was determined upon the results of the χ^2^ test and *I*^2^ test for heterogeneity. An *I*^2^ value of 50% or more indicated a substantial level of heterogeneity. Sensitivity analysis was applied to detect potential interference to the pooled effect size.

## Results

**Figure 1:**
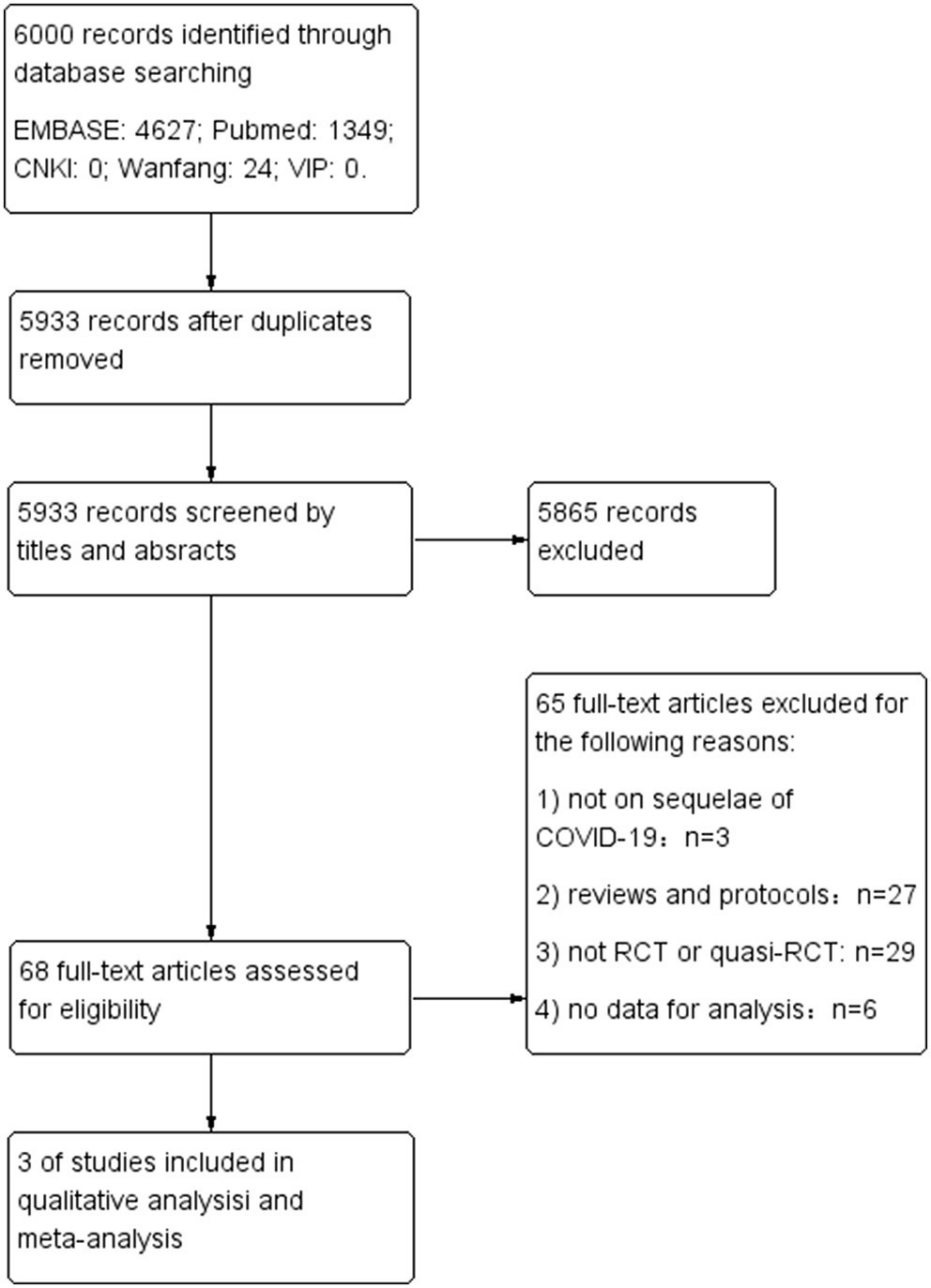
Flow diagram of study selection process.

The initial search identified 6,000 studies from 5 databases. Then 67 studies were removed as duplication. After title and abstract screening, 68 studies were selected and reviewed in full texts. Ultimately, three studies met the eligibility criteria and were included in the review (15–17). Details of the search and selection process are shown in Figure 1.

### Risk of Bias Assessment

**Figure 2:**
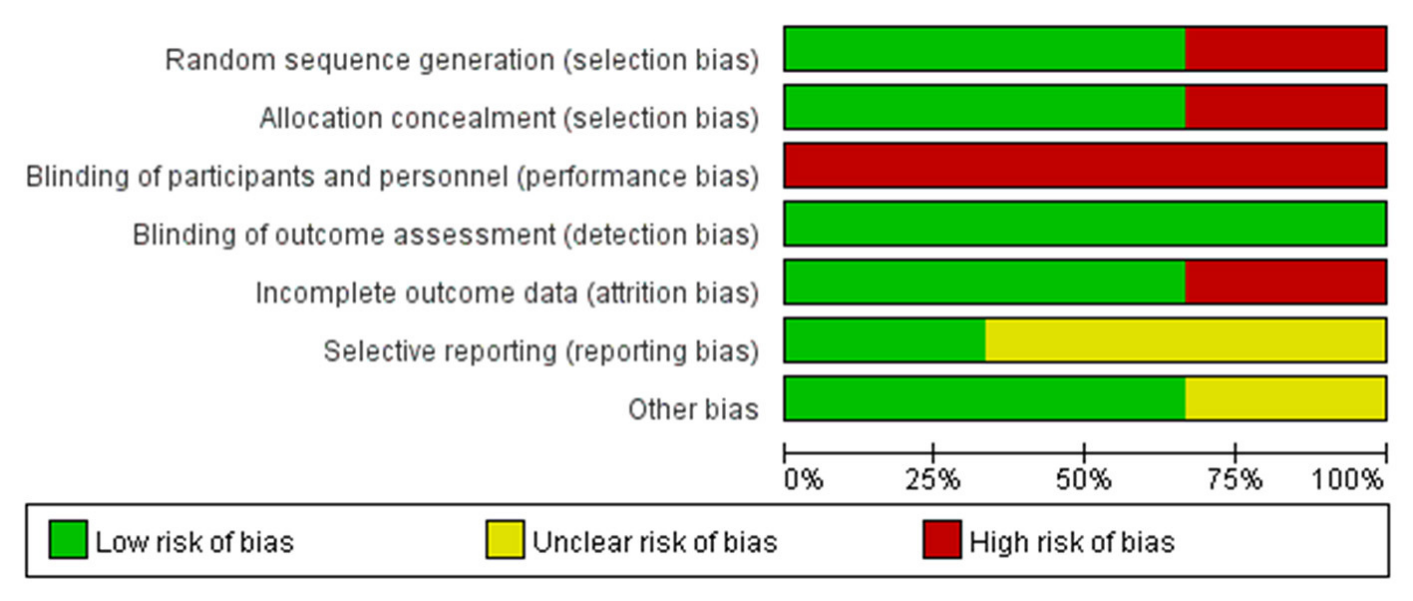
Risk of bias graph: review authors' judgements about each risk of bias item presented as percentages across all included studies.

**Figure 3:**
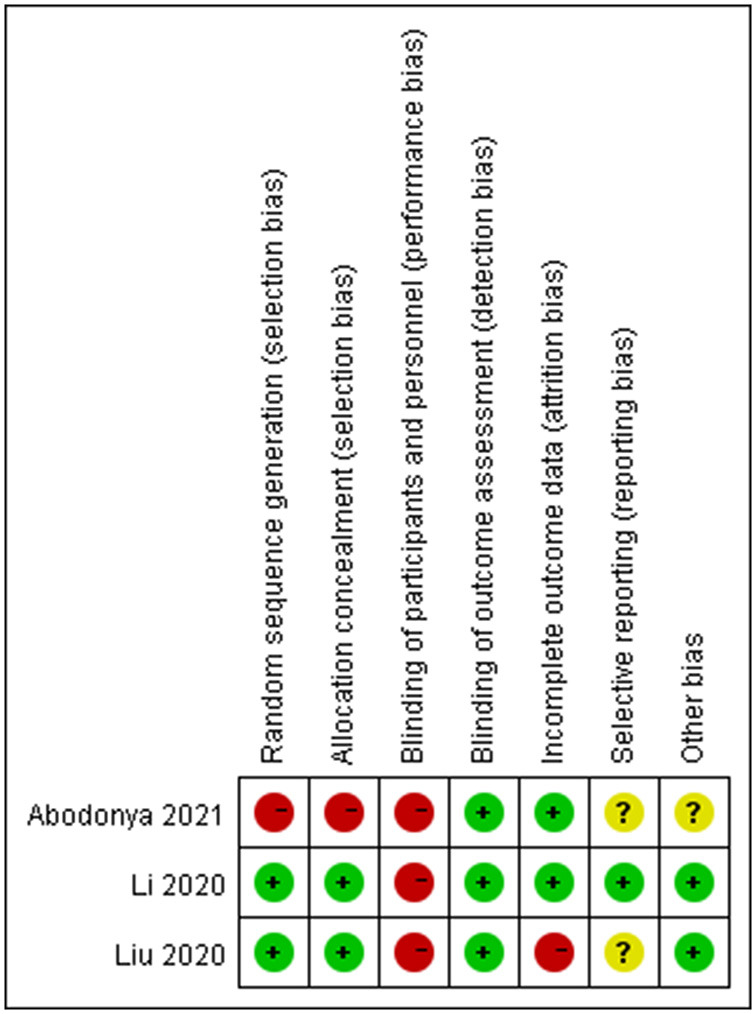
Risk of bias summary: review authors' judgements about each risk of bias item for each included study.

A study by Li reported details of the use of a computer-generated 1:1 block randomization (block size 10–14) stratified by hospital, as well as allocation concealment process (17). A study by Liu reported the use of odd/even number allocation generated by computer but not on how the allocation was concealed (16). Without randomization and details of allocation, a study by Abodonya evenly distributed their patients in two groups due to ethical considerations, which may introduce a high risk of bias (15). Due to the features of the interventions, none of the three studies was able to blind patients and personnel who provided the intervention, however, all of them reported the blinding of outcome assessors with varying details (15–17). A study by Li provided sufficient details on how the missing data were imputed. A study by Liu did not include missing data in the final analysis, and a study by Abodonya only mentioned that there was no loss-to-follow-up case. The risks of selective reporting in Liu and A study by Abodonya study were unclear as their protocols were not available to identify any unreported outcome. While a study by Li published their protocol in advance, which reported consistent outcome measurements with the RCT included in this review. The sample size of a study by Abodonya was based on availability rather than rigorous statistical calculation, which may undermine the estimation of effect size. No other bias was identified from the three studies (Figures 2, 3, produced by Review Manager 5.4.1.).

### Characteristics of Included Studies

Among the three included studies, two were RCTs from China, and one was a controlled trial from Saudi Arabia (15–17). The details of the included studies are shown in Table 1.

**Table 1:** Characteristics of included studies.

**References Study design**	**Sample size**	**Age (years)** **Mean *(*SD)**	**Inclusion criteria**	**ICU/MV history**	**Co-morbidity**	**Comparison groups**	**Treatment regimen**	**Frequency**	**Total session/ duration/ follow-up**	**Outcomes**
Liu et al. (16) China 2020 RCT	36	69.4 (8.0)	1. A definite diagnosis of COVID-19; 2. Aged ≥ 65 years; 3. 6 months after the onset of other acute diseases; 4. MMSE score > 21; 5. No COPD or any other respiratory disease; 6. FEV1 in 1s ≥70%.	NR	Hypertension T2MD osteoporosis	E: respiratory rehabilitation	Respiratory muscle training(device-based: threshold PEP); Cough exercise; diaphragmatic training; stretching exercise; home exercise.	10 min/ session, 2 sessions/ week	6 weeks	1. Pulmonary function (FEV1, FVC, FEV1/FVC, DLCO) 2. Exercise capacity (6-MWT) 3. QoL (SF-36) 4. Activities of daily living (FIM scale) 5. Anxiety and depression assessment(SDS, SAS)
	36	68.9 (7.6)				C: no care	N/A	N/A	N/A	
Abodonya et al. (15) Saudi Arabia 2021 quasi-RCT	21	48.3 (8.5)	1. Negative COVID; 2. Hemodynamically stable; 3. Respiratory rate <25breath/min; 4. Negative inspiratory force <25 cm H2O; 5. Minute ventilation <10L/min; 6. PO2/FIO2 > 200.	All admitted in ICU (mean length of MV 13.3 ± 7.6d/ 12.9 ± 8.4d)	NR	E: IMT+IBE	6 inspiratory cycles with 5min of resisted inspiration, followed by 60-second rest time in each cycle (device-based: threshold PEP)	2 sessions/ day, 5 days/week	20 sessions/ 2 weeks	1. Pulmonary function (FEV1, FVC, DSI) 2. Exercise capacity (6-MWT) 3. QoL (EQ-5D-3L) 4. Dyspnea severity index (DSI)
	21	47.8 (9.2)				C: IBE	NR	2 times daily	14 times/ 2 weeks	
Li et al. (17) China 2020 RCT	59	49.2 (10.8)	1. Discharged from one of the participating hospitals after inpatient treatment for COVID-19; 2. mMRC dyspnea score of 2–3.	86.6% with Oxygen support or non-invasive ventilation	Heart disease Hypertension Diabetes Obesity Lung disease (including Inactive TB) Others	E: TERECO+ education	Breathing control and thoracic expansion, aerobic exercise, LMS exercises specified in a 3-tiered exercise plan with difficulty and intensity scheduled to increase over time. +short education as control	40–60 mins/session, 3–4 sessions/ week + teleconsultations once/week	18-24 sessions/ 6 weeks, follow up for 28 weeks	1. Exercise capacity (6-MWT) 2. LMS (static squat test) 3. Pulmonary function (FEV1, FVC, FEV1/FVC, MVV, PEF) 4. QoL (SF-12) 5. Perceived dyspnea (mMRC) 6. Adverse events
	60	52.0(11.1)				C: education	10-min standardized educational Instruction on exercise, life-style, basic hygiene	Once at baseline	follow up for 28 weeks	

*MV, mechanical ventilation; E, experiment group; C, control group; MMSE, mini-mental state examination; T2DM, type 2 diabetes mellitus; PEP, positive expiratory pressure; FEV1, forced expiratory volume in one second; FVC, forced vital capacity; DLCO, diffusing lung capacity for carbon monoxide; 6-MWT, 6-min walk test; FIM, Functional Independence Measure; QoL, quality of life; SF-36, Short Form Health Survey-36; SDS, self-rating depression scale; SAS, self-rating anxiety scale; IMT, inspiratory muscle training; IBE, incentive spirometer exercise; Eq-5D-3L, EuroQuality-5Dimensions-3Levels questionnaire; DSI, dyspnea severity index; mMRC dyspnea score, modified British Medical Research Council dyspnea score; TERECO, tele-rehabilitation program for COVID-19; LMS, lower limb muscle strength; MVV, maximum voluntary ventilation; PEF, peak expiratory flow; SF-12, Short Form Health Survey-12; NA, not applicable; NR, not reported*.

#### Population and Enrollment

A total number of 233 patients with various degrees of lung impairments after COVID-19 were enrolled in the three studies with sample sizes ranging from 42 to 119. The studies included 135 males and 98 females with mean age varied from 47.8 ± 9.2 to 68.9 ± 7.6 years. With a negative result of COVID-19 after treatment, patients were recruited in a study by Li study with a mean interval of 70.07 ±16.85 days since discharged from hospital, while patients were recruited in ICU when weaned from MV in a study by Abodonya (15, 17). A study by Liu study recruited patients after discharge from hospital; however, the author of this study cannot be reached for details on the time interval between discharge and enrollment (16). In terms of the pulmonary function of the patient at the time of enrollment, mild-to-moderate lung function impairments were reported among patients according to inclusion criteria and baseline data provided by the three studies. Apart from a study by Abodonya, the other two studies reported comorbidities of patients observed at baseline, including mild-to-moderate heart disease, hypertension, diabetes, obesity, lung disease, and osteoporosis. All the three studies claimed that the baseline characteristics of patients were not statistically different between the experiment and control groups (15–17).

#### Details of Interventions

Although varied in details, rehabilitation interventions of the experiment groups in the three studies all employed respiratory muscle training, with or without endurance training (15–17). Two studies adopted device-based threshold positive expiratory pressure (threshold PEP), which can increase airway diameter and enhance mucus clearance by generating a force for flow obstruction that allows flow only when PEP reaches the requisite threshold level (15, 16, 18). One study also applied lower limb muscle strength (LMS) exercises to improve muscle mass and strength, and one study integrated stretching exercise for body posture and flexibility (16, 17). All the three studies adopted the interval training approach (15–17).

Patients randomized to the experiment groups in studies by Liu and Abodonya conducted their exercises under supervision or assistance of specialized therapists or doctors in a face-to-face manner in hospital, while patients in a study by Li followed an unsupervised exercise program delivered by a smartphone application at home, with a teleconsultation once a week. In terms of the control group, a study by Liu compared rehabilitation intervention with no treatment, while the other two studies chose incentive spirometer exercise and short education as comparators, respectively (15, 17).

Two studies conducted their therapies in a 6-week duration with varying frequency and schedule, while the other study delivered the program in 2 weeks for patients (15–17). Only a study by Li followed up with their patients for 22 weeks after completion of treatment. A study by Li did not find any serious adverse events occurred during study period, and the other two studies did not report on adverse events (15–17).

### Effect of Interventions

Details of outcomes of included studies are shown in Table 2.

**Table 2:** Summary of outcomes.

**References**		**Outcome measurement**	**Experiment group**			**Control group**				**Differences of changes**	
			**Baseline** **(mean ±SD)**	**After-PR** **(mean ±SD)**	**Changes** **(mean ±SD)**	**Baseline** **(mean ±SD)**	**After-PR** **(mean ±SD)**	**Changes** **(mean ±SD)**	**RR/MD(95%CI)**	***P*-value**	***P*-value^**#**^**
Liu et al. (16)	Exercise capacity	6-MWT, m	162.7 ± 72.0	212.3 ± 82.5	49.6 ± 85.08^*^	155.7 ± 82.1	157.2 ± 71.7	1.50 ± 84.69^*^	48.1 (8.89,87.31)^*^	<0.05	<0.05
	Pulmonary function	FEV1, L	1.10 ± 0.08	1.44 ± 0.25	-	1.13 ± 0.14	1.26 ± 0.32	-	-	<0.05	-
		FVC, L	1.79 ± 0.53	2.36 ± 0.49	-	1.77 ± 0.64	2.08 ± 0.37	-	-	<0.05	-
		FEV1/FVC, %^**^	60.48 ± 6.39	68.19 ± 6.05	-	60.44 ± 5.77	61.23 ± 6.43	-	-	<0.05	-
		DLCO, % pred	60.3 ± 11.3	78.1 ± 12.3	-	60.7 ± 12.0	63.0 ± 13.4	-	-	<0.05	-
	QoL (SF-36)	Physical health	52.4 ± 6.2	71.6 ± 7.6	-	53.2 ± 7.7	54.1 ± 7.5	-	-	<0.05	-
		Body role function	61.2 ± 6.6	75.9 ± 7.9	-	61.3 ± 7.2	62.0 ± 7.3	-	-	<0.05	-
		Physical pain	63.5 ± 7.4	78.3 ± 7.8	-	63.5 ± 8.1	62.9 ± 7.9	-	-	<0.05	-
		General health	61.8 ± 7.7	74.2 ± 7.9	-	61.8 ± 8.4	61.4 ± 6.9	-	-	<0.05	-
		Energy	60.6 ± 6.9	75.6 ± 7.1	-	60.5 ± 7.1	61.2 ± 6.3	-	-	<0.05	-
		Social function	59.4 ± 7.2	69.8 ± 6.4	-	59.5 ± 7.0	58.9 ± 6.6	-	-	<0.05	-
		Emotional role function	61.4 ± 6.9	75.7 ± 7.0	-	61.4 ± 7.3	60.8 ± 7.3	-	-	<0.05	-
		Mental health	61.5 ± 6.5	73.7 ± 7.6	-	61.6 ± 7.2	62.1 ± 7.6	-	-	<0.05	-
	ADL	FIM	109.2 ± 13.0	109.4 ± 11.1	-	109.3 ± 10.7	108.9 ± 10.1	-	0.50 ± 2.50	>0.05	
	Anxiety & depression	SAS score	56.3 ± 8.1	47.4 ± 6.3	-	55.8 ± 7.4	54.9 ± 7.3	-	-	<0.05	-
		SDS score	56.4 ± 7.9	54.5 ± 5.9	-	55.9 ± 7.3	55.8 ± 7.1	-	-	>0.05	-
Abodonya et al. (15)	Exercise capacity	6-MWT, m	332.6 ± 34.5	376.5 ± 39.4	43.9 ± 40.68^*^	329.7 ± 37.8	334.8 ± 38.2	5.1 ± 41.41^*^	38.8 (13.97,63.63)^*^	=0.028	<0.05
	Dyspnea	DSI	18.5 ± 4.3	14.2 ± 3.5	-	17.8 ± 5.1	17.1 ± 4.8	-	-	=0.032	-
	Pulmonary function	FEV1, % pred	76.2 ± 12.7	83.7 ± 10.5	-	75.4 ± 12.2	75.1 ± 12.4	-	-	=0.043	0.06
		FVC, % pred	78.7 ± 13.5	84.2 ± 10.3	-	77.2 ± 12.6	76.8 ± 11.7	-	-	=0.041	-
	QoL	Eq-5D-3L	38.6 ± 5.8	59.4 ± 8.3	-	40.7 ± 6.2	43.3 ± 6.5	-	-	=0.021	-
Li et al. (17)	Exercise capacity	6-MWT, m	514.52 ± 82.87	-	80.20 ± 74.66	499.98 ± 93.41	-	17.09 ± 63.94	65.45 (43.80, 87.10)	<0.001	-
	LMS	Squat time, s	34.68 ± 21.85	-	29.35 ± 27.22	38.60 ± 25.07	-	7.98 ± 19.53	20.12 (12.34, 27.90)	<0.001	-
	Perceived dyspnea	mMRC, %^†^	-	-	90.4	-	-	61.7	1.46 (1.17, 1.82)	=0.001	-
	Pulmonary function	FEV1, L	2.24 ± 0.74	-	0.28 ± 0.51	2.14 ± 0.69	-	0.18 ± 0.53	0.08 (−0.08, 0.25)	=0.327	-
		FEV1, % pred	79.10 ± 18.25	-	-	77.95 ± 15.45	-	-	-	-	-
		FEV1 below LLN, n (%)	26 (44.8)	-	-	24 (42.1)	-	-	-	-	-
		FVC, L	2.85 ± 0.75	-	0.21 ± 0.47	2.69 ± 0.87	-	0.19 ± 0.40	0.02 (−0.14, 0.18)	=0.818	-
		FVC, % pred	83.62 ± 14.99	-	-	80.43 ± 15.39	-	-	-	-	-
		FVC below LLN, n (%)	23 (39.7)	-	-	22 (38.6)	-	-	-	-	-
		FEV1/FVC	0.79 ± 0.14	-	0.04 ± 0.17	0.81 ± 0.12	-	0.01 ± 0.16	0.03 (−0.02, 0.07)	=0.224	-
		FEV1/FVC, % pred	95.03 ± 16.78	-	-	97.9 ± 15.0	-	-	-	-	-
		FEV1/FVC below LLN, n (%)	14 (24.1)	-	-	12 (21.1)	-	-	-	-	-
		MVV, L/min	74.3 ± 30.6	-	14.5 ± 21.6	63.05 ± 26.12	-	5.61 ± 17.3	10.57 (3.26, 17.88)	=0.005	-
		MVV, % pred	66.37 ± 22.88	-	-	58.94 ± 20.86	-	-	-	-	-
		PEF, L/s	4.21 ± 2.33	-	0.98 ± 1.90	3.66 ± 1.75	-	0.66 ± 1.95	0.38 (−0.24, 1.00)	=0.229	-
		PEF, % pred	51.42 ± 24.70	-	-	46.41 ± 18.20	-	-	-	-	-
	QoL (SF-12)	PCS	39.15 ± 7.16	-	7.81 ± 7.02	39.69 ± 7.06	-	3.84 ± 7.60	3.79 (1.24, 6.35)	=0.004	-
		MCS	44.67 ± 8.76	-	6.15 ± 10.78	44.13 ± 8.25	-	4.17 ± 8.79	2.18 (−0.54, 4.90)	=0.116	-

* *Changes within group(post-treatment value minus baseline value) and between groups (mean difference of change between groups, treatment effect) were calculated based on data provided in the included studies using R software*;

# *P value was from our analysis*;

** *FEV1/FVC ratio was presented in percentage in Liu's study*;

† *the mMRC dyspnea score in the original paper was transformed to dichotomous variable, favorable outcome (mMRC score = 0, coded 1 in analysis), and non-favorable outcome (all other mMRC scores, coded 0 in analysis). PR, pulmonary rehabilitation; FEV1, forced expiratory volume in one second; % pred, % predicted; FVC, forced vital capacity; DLCO, diffusing lung capacity for carbon monoxide; 6-MWT, 6-minute walk test; QoL, quality of life; SF-36, Short Form Health Survey-36; ADL, Activities of daily living; FIM, Functional Independence Measure; SDS, self-rating depression scale; SAS, self-rating anxiety scale; Eq-5D-3L, EuroQuality-5Dimensions-3Levels questionnaire; DSI, dyspnea severity index; LLN, lower limit of normal; MVV, maximum voluntary ventilation; PEF, peak expiratory flow; LMS, lower limb muscle strength; SF-12, Short Form Health Survey-12; PCS, physical component score; MCS, mental component score; mMRC dyspnea score, modified British Medical Research Council dyspnea score*.

#### Exercise Capacity

All three studies reported results from 6-min walking test (6-WMT) to demonstrate improvement made on exercise capacity, however, large variation was observed across baseline data on 6-WMT.

A study by Liu reported that distance of 6-MWT was significantly longer in experiment group than that in control group at 6 weeks from baseline (212.3 ± 82.5 vs. 157.2 ± 71.7; *p* < 0.05), and the improvement on 6-MWT was also significant within experiment group (162.7 ± 72.0, 212.3 ± 82.5; *p* < 0.05) but not in control group (155.7 ± 82.1, 157.2 ± 71.7; *p* > 0.05). We calculated the change of 6-MWT through 6 weeks of PR within each group, and found consistent results with the original study, and the between-group difference in change of 6-WMT (48.1, 95%CI 8.89 to 87.31; *p* < 0.05) was superior to the minimal clinical important difference (MCID) of 30 meters recommended for the 6-MWT in chronic lung disease, given MCID for COVID-19 has not yet been established (19).

In a study by Abodonya, the between-group difference in change of 6-WMT was statistically significant at 2 weeks from baseline in our calculation (38.8, 95%CI 13.97 to 63.63; *p* < 0.05), and was also superior to MCID of 30 meters (19). Within each group, the experiment group showed a significant increase after the intervention (332.6 ± 34.5, 376.5 ± 39.4, change 43.9 ± 40.68; *p* < 0.001), whereas, the control group showed non-significant change (329.7 ± 37.8, 334.8 ± 38.2, change 5.1 ± 41.41; *p* = 0.624).

As primary outcome, a study by Li reported that 6-MWT was improved in both the experiment (80.20 ± 74.66) and control group (17.09 ± 63.94) over 6 weeks of intervention, and the difference in change of 6-MWT at 6 weeks from baseline was statistically significant between the two groups (65.45, 95%CI 43.80 to 87.10; *p* < 0.001), and was more than double the recommended MCID (19).

**Figure 4:**
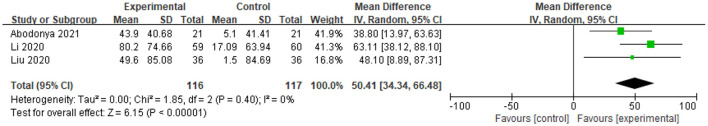
Mean difference of change on 6-MWT between 3 studies after intervention from baseline.

We synthesized the difference in change of 6-MWT between groups from all three studies, and found that the pooled estimate of effect of PR on 6-MWT (MD 50.41, 95% CI 34.34 to 66.48; *p* < 0.0001) was in favor of experiment group, and also superior to the recommend MCID (19). Sensitivity analysis was conducted by moving a study by Abodonya with the smallest sample size, and a study by Li with endurance exercise, the direction of the pooled estimate did not change (Figure 4).

A study by Li also tested the effect of PR on lower LMS with squat time, reporting that the experiment group made statistically significant improvement on squat time than the control group did (20.12, 95% CI 12.34 to 27.90; *p* < 0.001), although an improvement on squat time can be seen in both the groups (17).

#### Dyspnea

A study by Abodonya reported on dyspnea using DSI, with a high score indicating worsened severity of dyspnea. It was found that the DSI score was significantly decreased in the experiment group (18.5 ± 4.3, 14.2 ± 3.5) than that in the control group (17.8 ± 5.1, 17.1 ± 4.8) at 2 weeks from baseline (*p* = 0.032) (15).

A study by Li reported on patient-perceived dyspnea using mMRC dyspnea score. The mMRC dyspnea score in the original paper was transformed to the dichotomous variable (mMRC score ≠ 0 as favorable outcome, mMRC scores ≠ 0 as non-favorable outcome), and presented as percentage of patients with favorable outcomes in each group. It was found that the RR of patients with favorable outcomes was 1.46 (95% CI 1.17 to 1.82; *p* = 0.001) between experiment and control groups at 6 weeks from baseline (17).

#### Pulmonary Function Tests

Pulmonary function, commonly described by spirometry (e.g. FEV1, FVC, FEV1/FVC ratio, and their % predicted values, etc.), lung volumes (TLC, etc.), and diffusion capacity (DLCO and % predicted, etc.), was designed as a primary outcome in two studies, but presented by all the three included studies with various parameters (6, 15, 16).

A study by Liu reported FEV1, FVC, FEV1/FVC ratio, and DLCO % predicted at baseline and after the intervention. Significant differences were detected between experiment and control groups at 6 weeks from baseline on FEV1 (1.44 ± 0.25 vs. 1.26 ± 0.32; *p* < 0.05), FVC (2.36 ± 0.49 vs. 2.08 ± 0.37; *p* < 0.05), FEV1/FVC (presented in percentage, 68.19 ± 6.05 vs. 61.23 ± 6.43; *p* < 0.05), DLCO % predicted (78.1 ± 12.3 vs. 63.0 ± 13.4; *p* < 0.05), respectively. Meanwhile, the improvements on all the above parameters were also significant within experiment group through 6 weeks of PR (all p < 0.05), but not in the control group (16).

A study by Abodonya only reports values of FEV1 % predicted and FVC % predicted. When compared between groups, it showed that the experiment group demonstrated significantly more changes than the control group did on FEV1 % predicted (*p* = 0.043) and FVC% predicted (*p* = 0.041) at 2 weeks from baseline (completion of the intervention), respectively. Within each group, changes on FEV1% predicted (76.2 ± 12.7, 83.7 ± 10.5; *p* = 0.047) and FVC% predicted (78.7 ± 13.5, 84.2 ± 10.3; *p* = 0.039) were reported as significant at 2 weeks from baseline in experiment group, however, they seemed not significant in the control group (*p* = 0.87; *p* = 0.754, respectively) (5).

A study by Li reported a series of results of PFT, including FEV1, FVC, FEV1/FVC, FVC, maximum voluntary ventilation in liters per minute (MVV) and peak expiratory flow (PEF), and their percentage of predicted value and number (%) below lower limit of normal (LLN). It reported that lung function parameters improved in both groups over time, however, no significant between-group difference was found apart from an adjusted between-group difference in change of MVV at 6 weeks from baseline (10.57 L/min, 95% CI 3.26 to 17.88; *p* = 0.005) in favor of the experiment group. Moreover, it was found that the changes of FEV1 (0.28 ± 0.51, 12.5% of baseline) and FVC (0.21 ± 0.47, 7.4% of baseline) in the experiment group did not exceed the clinically meaningful change threshold (week to week) for patients with COPD recommended by ATS/ERS (17).

Considering the observed heterogeneity on starting time of PR, intervention design and outcome parameters, inconsistent results from PFT, as well as potential statistical bias that may be introduced, we did not synthesize the data on between-group differences in changes of FEV1, FVC, and FEV1/FVC after intervention from baseline in the three studies.

#### Quality of Life

All three studies reported the effect of PR on QoL, using varied inventories, including SF-12, SF-36, and Eq-5D-3L.

A study by Liu adopted SF-36 with eight domains to measure the effect of intervention on QoL of patient. Scores were significantly higher in experiment group than that in control group at 6 weeks from baseline in all domains (physical health: 71.6 ± 7.6, vs. 54.1 ± 7.5, *p* < 0.05; body role function: 75.9 ± 7.9 vs. 62.0 ± 7.3, *p* < 0.05; physical pain: 78.3 ± 7.8 vs. 62.9 ± 7.9, *p* < 0.05; general health: 74.2 ± 7.9 vs. 61.4 ± 6.9, *p* < 0.05; energy: 75.6 ± 7.1 vs. 61.2 ± 6.3, *p* < 0.05; social function: 69.8 ± 6.4 vs. 58.9 ± 6.6, *p* < 0.05; emotional role function: 75.7 ± 7.0 vs. 60.8 ± 7.3, *p* < 0.05; mental health: 73.7 ± 7.6 vs. 62.1 ± 7.6, *p* < 0.05), and the improvements on all scores through 6 weeks were also statistically significant within experiment group (all *p* < 0.05). The study reported activity of ADL with the Functional Independence Measure (FIM) scale, which contains 18 items on motor, communication, and social cognition. However, no significant difference was found within and between groups (*p* > 0.05) (16).

A study by Abodonya used Eq-5D-3L to assess QoL and reported that the overall score of Eq-5D-3L was significantly higher in the experiment group than that in the control group at 2 weeks from baseline (59.4 ± 8.3 vs. 43.3 ± 6.5; *p* = 0.021). Within each group, the overall score was significantly improved in PR group through 2 weeks (38.6 ± 5.8 vs. 59.4 ± 8.3; *p* < 0.001), but not in the control group (40.7 ± 6.2, 43.3 ± 6.5, p = 0.173) (15).

The Short Form Health Survey-12 (SF-12) was applied in a study Li to measure the physical and mental status of patients. It reported that the difference in change was significant on the score of physical component between groups (3.79, 95% CI 1.24 to 6.35; *p* = 0.004) at 6 weeks from baseline, but not significant on the score of mental component (2.18, 95% CI 0.54 to 4.90; p = 0.116) (17).

#### Anxiety and Depression

A study by Liu assessed the anxiety and depression status of patients using self-rating depression scale (SDS) and self-rating anxiety scale (SAS). Given the similarity of baseline characteristics between the two groups, it was found that the SAS score in the experiment group was significantly lower than that in the control group at 6 weeks from baseline (47.4 ± 6.3 vs. 54.9± 7.3; *p* < 0.05), the decrease of SAS score within experiment group at 6 weeks from baseline was also significant (56.3 ± 8.1 vs. 47.4 ± 6.3; *p* < 0.05). However, the SDS scores were not significantly different between the two groups at 6 weeks from baseline (*p* > 0.05) (16).

## Discussion

The three studies included in this review adopted respiratory muscle training, with or without endurance training. The pooled estimate of PR effect on 6-MWT among three studies demonstrated the PR could improve the exercise capacity with clinical importance for patients with post-COVID-19. Although using diverse assessment tools, the three studies showed that PR could improve the symptom of dyspnea and QoL for patients survived from COVID-19. However, results of the PR effects on pulmonary function (i.e. FEV1, FVC, FEV1/FVC, etc.) were contradictory across studies. No severe adverse event was reported by the three studies. The risk of bias among the three studies also varied, with major concerns on the risk of blinding of participants and interventions performers, as well as incomplete data reporting.

With a considerable variation on baseline data of 6-MWT, all the three studies reported respiratory muscle training significantly improved exercise capacity, regardless of the types of interventions (face-to-face or remote, with device-based or not, with endurance training or not). Evidence suggested that pulmonary interstitial changes from CT images can be observed among patients with COVID-19, and lasted over 12 months after discharge (20–22). Although the mechanisms of respiratory limitation in COPD and interstitial lung disease (ILD) differ, the similarities in clinical problems (exercise intolerance, muscle dysfunction, dyspnea, impaired QoL) suggest that PR may also benefit patients with ILD (10). Previous RCTs demonstrated that benefits of PR were smaller on functional exercise tolerance, dyspnea, and QoL in patients with ILD compared with that in patients with COPD, and were yet fading 6 months after training (23–25). The pooled estimate of the difference in change of 6-MWT (50.41 meters) between groups in this review had clinical importance based on a MCID of 30 meters recommended for chronic lung disease (19). However, this was comparable to the difference of 56.7 meters reported by a RCT on a 6-week outpatient exercise program for SARS survivors in Hong Kong, and the difference of 43.9 m reported by a review of McCarthy Cochrane on PR for patients with COPD (26, 27). Such results from previous studies may be associated with the much severer degree of lung impairment observed in a review by McCarthy (27). It is worth noting that two out of three studies in this review did not integrate endurance exercise training, which primarily focuses on improving exercise capacity (10). Therefore, it is possible that the effect size on 6-MWT would be amplified if all the three studies added endurance training in their programs. However, the long-term effect of PR on 6-MWT for patients with post-COVID-19 remained unconvinced at this stage. Although a lasting benefit of PR on 6-MWT at 22 weeks after training was reported in a study by Li, no further follow-up data were available in the review, and the effect may not sustain longer, as previous studies found the gains in inspiratory muscle function were lost 12 months after cessation of the inspiratory muscle training (IMT) program (28).

Many factors may affect the effect size of PR on 6-MWT across studies included in this review. The relatively long distance of 6-MWT observed in a study by Li, compared with other two included studies, was likely attributable to the aerobic exercise (endurance training) as an adjunct to IMT, its outdoor setting of the walking test and better exercise capacity of patients at baseline, rather than the method (i.e. telerehabilitation or face-to-face PR) that the training was delivered (10, 29, 30). In terms of timing of PR, evidence showed that early initiation of PR (shortly after hospitalization or as early as during acute or critical illness) for patients with COPD was clinically effective and safe and could hasten recovery (10). However, it was hard to judge whether the difference on 6-MWT observed across studies was statistically significant and associated with varied time intervals between hospital discharge and commencement of PR. Meanwhile, given the possibility of simultaneous recovery of lung function after COVID-19 and lack of no treatment control, it was not possible to evaluate the true effect size of PRs on 6-MWT in the three studies in short- or long-term beyond 28 weeks from baseline.

Reduction of dyspnea, one of the most common symptoms among individuals with chronic respiratory disease, is an important target of PR (10, 31). Similar to previous RCTs of PR in patients with ILD, a study by Li reported an unsustainable effect on perceived dyspnea using mMRC dyspnea score and explained the effect as objective due to cognitive need of patients to change during the intervention (23–25, 32). A review by Cox on telerehabilitation for chronic respiratory disease detected little or no difference for breathlessness using Chronic Respiratory Questionnaire (CRQ) dyspnea domain score (29). Whereas, two systematic reviews and meta-analyses of IMT in patients with COPD with severer lung impairment demonstrated significant and clinically meaningful reductions in dyspnea during ADL, using CRQ dyspnea score or transitional dyspnea index (27, 33). Unlike endurance exercise training, IMT confers limited improvement on dyspnea or maximal exercise capacity (33–36). Instead, it has an additional benefit on inspiratory muscle strength and endurance, which may benefit individuals with marked inspiratory muscle weakness (10). Apart from variations in design of PR program, controls, and timing of intervention, diversity in assessment tools of dyspnea may also explain the difference of PR's effect on dyspnea observed. As the perception of the sensation of an individual, dyspnea can be difficult to measure. Therefore, a recommended list of tools, taking account of the type of PR and short- and long-term effects, should be developed for future research studies.

Similar situation was found in assessment of QoL in this review. Various assessment tools and contradictory results were seen across studies, making it difficult for data synthesis. Using SF-36, the Hong Kong study did not detect any effect of exercise training on QoL in contrast with a study by Liu (16, 26). A RCT on device-based respiratory muscle training for patients with COPD detected non-significant difference in QoL between groups with a clinical COPD questionnaire (CCQ), whereas a review by McCarthy reported a statistically significant improvements of QoL measured by the St. George's Respiratory Questionnaire (SGRQ) and CRQ among patients with COPD (27, 37). Anxiety and depression were evaluated separately in a study by Liu, while in most of the case emotion was assessed as part of qualify of life. Further evidences are needed for a robust conclusion on PR's effect on QoL.

Most up-to-data clinical data and systematic review revealed that a considerable proportion (22–56% across different severity scales) of patients had a pulmonary diffusion abnormality 6 months after symptom onset, compared with restrictive pattern (15%) and obstructive pattern (7%) (6, 7). The finding was confirmed by the pulmonary interstitial changes found from autopsies and CT images of patients with COVID-19 (20, 38). However, only a study by Liu reported before- and after-treatment data on DLCO % predicted, which was closely associated with the diffusion capacity of patients. Even if the reported difference in change of DLCO % predicted was significant between groups in a study by Liu, the value in the experiment group was close to but still under the borderline of normal after 6 weeks of intervention, if a 80% cutoff point was adopted as other studies did (2, 6, 7). The remaining gap between DLCO % predicted and the 80% cutoff point may be explained by chance or remaining impairment after rehabilitation.

As essential parameters for the ventilation capacity of patients, FEV1and FVC in various formats (e.g., absolute value, ratio, % predicted value, etc.) were presented but with conflicting results from the three studies. As a method of categorizing the severity of lung function, FEV1 % predicted was improved significantly by the device-based IMT in a study Abodonya, however, the result was questionable due to major weakness on study design (15, 39). In terms of the absolute value of FEV1, FVC, and FEV1/FVC ratio, the interpretation should be done with caution as inconsistent results were reported in studies by Liu and Li. Similar to a study by Liu, a recent systematic review and meta-analysis also reported superior effects of breathing exercise on lung function parameters (FEV1 and FEV1/FVC) as compared with control for COPD (40). The divergence observed between studies may be explained by the difference in design and intensity of interventions, which may not sufficiently target on lung function (17). The starting time point of intervention from hospital discharge (much longer interval in a study by Li) and the severity of COVID-19, age, and comorbidities at enrollment may also contribute to the differences in FEV1 and FEV1/FVC (16, 17). In addition, MVV was not discussed separately in this review, due to the good correlation with FEV1 and little additional contribution to diagnosis and follow-up evaluation in the clinical setting (24, 39).

There are many factors associated with the quality and interpretation of PFT. An important aspect to consider is the time to perform such tests. The British Thoracic Society (BTS) recommended the evaluation of PFTs at 3 months post-discharge, especially for patients suspected of having an interstitial disease (41). However, two of the studies did not follow this recommendation, and one study had follow-up at 28 weeks from baseline reported unsustainable effects on some of the parameters after primary follow-up (17). This may lead to problematic estimates of effect size either in short or long term. Highlighted by ATS/ERS in a number of guidelines, the LLN should be used as the reference for the definition of normal in a certain population rather than a fixed cutoff point (e.g., 0.7 as a lower limit for FEV1/FVC ratio) in order to avoid false-positive result (39, 42–44). The numbers (%) below LLN for FEV1, FVC, and FEV1/FVC were not reported in most of the studies included and their reference of the population was different. Therefore, it can be arbitrary to estimate the real effect size and interpret the change observed on parameters of lung impairment among patients, using absolute value and/or fixed cutoff point. In addition, comorbidity of the patient is another important factor to consider when PR is conducted and measured, and a thorough evaluation of its impact on PR's effect is necessary when data are available.

Along with increasing number of RCTs published, this review was able to summarize and present the effect of PRs through multiple dimensions, which became the major strength of this review, whereas limitations came from the following aspects. First, the number of studies included in this review was scarce. Apart from IMT and endurance exercise training as an adjunct, no studies on other types of PRs were retrieved and evaluated due to data availability. In addition, the restrictions on study design may also limit the full understanding of the effect of individual PR. Due to the limited number of studies and heterogeneity across studies, the results from PFT were not synthesized, which may further restrict our estimation of the effect size of the intervention. Second, major flaws, such as problematic randomization and absence of blinding of patients and training performers in some of the included studies, may undermine the validity and reliability of results from individual study or data synthesis. Third, the review did not provide adequate evidence on the effect of PR for patients with severe lung impairment due to the population included (mild-to-moderate lung impairment), and long-term (>1 year) effect for PR among patients with post-COVID-19. Last but not the least, the review was not able to evaluate the effect of PR for comorbidity due to lack of data. Therefore, the interpretation and generalization of the result of this review should be cautious.

## Conclusion

This review showed that PR could improve exercise capacity measured by 6-MWT among patients with mild-to-moderate lung impairment associated with COVID-19. The interpretation of effects on lung function, dyspnea, and QoL should be cautious due to inadequate and conflicting data reported across studies. More rigorous and long-term evidence on the effect of PR among patients, especially those with severe lung impairment, are needed to guide the PR practice for the increasing number of survivors of COVID-19.

## Data Availability Statement

The original contributions presented in the study are included in the article/supplementary materials, further inquiries can be directed to the corresponding author/s.

## Author Contributions

HC and ZL contributed to the project development. HS, TS, HC, and JW contributed to the database searches, literature screening, and data extraction. HC, HS, and XL contributed to the data analysis. HC and XL contributed to the manuscript writing. JW and ZL contributed to the critical review of the manuscript. All authors approved the final version of this manuscript to be published and agree to be accountable for all the aspects of this study.

## Funding

This study was funded by the National Key Research and Development Plan (No. 2020YFC0845000) under the program of Clinical Evaluation of the Effect of Traditional Chinese Medicine on Convalescent Period of COVID-19 Infection.

## Conflict of Interest

The authors declare that the research was conducted in the absence of any commercial or financial relationships that could be construed as a potential conflict of interest.

## Publisher's Note

All claims expressed in this article are solely those of the authors and do not necessarily represent those of their affiliated organizations, or those of the publisher, the editors and the reviewers. Any product that may be evaluated in this article, or claim that may be made by its manufacturer, is not guaranteed or endorsed by the publisher.
